# PTEN: A Novel Diabetes Nephropathy Protective Gene Related to Cellular Senescence

**DOI:** 10.3390/ijms26073088

**Published:** 2025-03-27

**Authors:** Kang Li, Huidi Tang, Xiaoqing Cao, Xiaoli Zhang, Xiaojie Wang

**Affiliations:** 1 Department of Pharmacology, School of Basic Medical Sciences, Shandong University, Jinan 250012, China; 2 Department of Cardiology, Shandong Public Health Clinical Center, Shandong University, Jinan 250013, China; 3 Key Laboratory of the Ministry of Education for Experimental Teratology, Department of Histology and Embryology, School of Basic Medical Sciences, Shandong University, Jinan 250012, China

**Keywords:** diabetic nephropathy, WGCNA, clustering analysis, machine learning, *PTEN*

## Abstract

Diabetic nephropathy (DN) is the leading cause of end-stage renal disease (ESRD). The current diagnostic and therapeutic approaches need to be improved. Cellular senescence has been implicated in the pathogenesis of DN, but its precise role remains unclear. This study aimed to identify key pathogenic genes related to cellular senescence in DN and explore their potential as diagnostic biomarkers. Using transcriptomic data from GEO datasets (GSE96804, GSE30122, GSE142025, and GSE104948) and cellular senescence-related genes sourced from the GenAge database, we integrated multiple bioinformatics approaches, including differential expression analysis, weighted gene co-expression network analysis (WGCNA), machine learning and protein–protein interaction (PPI), to identify diagnostic genes. *PTEN* was identified as a key diagnostic gene. Immune infiltration analysis revealed that *PTEN* expression is positively correlated with macrophage M2 and dendritic cell resting infiltration and negatively correlated with monocytes and neutrophils. snRNA analysis revealed that *PTEN* is mainly expressed in mesangial cells. Finally, RT-PCR results revealed that the mRNA expression of *PTEN* was upregulated in kidneys from db/db *mice*. Additionally, high-glucose treatment significantly upregulated *PTEN* expression in cultured *human* mesangial cells. This study identifies *PTEN* as a potential diagnostic biomarker for DN which may contribute to early detection and personalized therapeutic strategies.

## 1. Introduction

Diabetic nephropathy (DN) is a major complication of diabetes, characterized primarily by hypertension, proteinuria, and progressive decline in renal function [1,2]. It is the leading cause of end-stage renal disease (ESRD) [3]. The fundamental pathological features of DN include tubulointerstitial lesions, thickening of the glomerular basement membrane, expansion of the mesangial matrix, and nodular glomerulosclerosis [4]. The diagnostic methods for DN are limited, and microalbuminuria is one of the key indicators used in clinical practice to assess DN progression [5]. However, not all patients with diabetes or renal failure exhibit significant proteinuria [6]. Currently, the gold standard for diagnosing DN is renal biopsy [7], an invasive procedure associated with complications such as infection and bleeding [8]. Current treatment strategies for DN involve controlling blood pressure and glucose levels and inhibiting the renin–angiotensin system (RAS) to slow DN progression [9,10]. For example, a variety of drugs, such as sodium-glucose cotransporter-2 inhibitors (SGLT2i) and glucagon-like peptide-1 (GLP-1), can be used for the treatment of DN. However, owing to the heterogeneity of DN among individuals, not all patients respond effectively to these treatments. Therefore, to enable the early diagnosis and treatment of DN, it is crucial to explore non-invasive, sensitive, and effective biomarkers. With rapid advancements in genomics and bioinformatics, numerous disease databases have been established and refined, providing a theoretical foundation for exploring novel therapeutic targets and the underlying mechanisms of diseases [11]. By utilizing various bioinformatics approaches, potential biomarkers for the diagnosis and treatment of DN have been identified [12,13]. For instance, in the study by Xu et al. [14], datasets related to DN from the Gene Expression Omnibus (GEO) database were analyzed using weighted gene co-expression network analysis (WGCNA), machine learning, and protein–protein interaction (PPI) networks, leading to the identification of *CD36*, *ITGB2*, and *SLC1A3* as highly correlated with DN, with excellent diagnostic performance. Bioinformatics holds tremendous potential for uncovering novel biomarkers in disease research.

Cellular senescence is a complex biological process involving alterations in gene expression, loss of protein function, and cell cycle arrest, ultimately leading to inflammation, impaired tissue repair, irreversible tissue damage, and organ dysfunction [15,16]. Under the stimulation of pathogenic factors such as hyperglycemia, senescent cells secrete senescence-associated secretory phenotype (SASP) components, which promote inflammation and contribute to a decline in renal function [17]. SASP includes cytokines, chemokines, and growth factors, and is considered a potential source of inflammatory mediators in DN [18]. Enhanced expression of senescence-associated β-galactosidase (SA-β-gal) is recognized as a hallmark biomarker of cellular senescence [19]. Increasing evidence suggests that the premature senescence of mesangial cells and podocytes is closely associated with DN [20]. Senescent cells have been identified in the kidneys of both patients with DN and experimental animal models [21,22]. Compared with *human* control tissues, the SA-β-gal staining and p16 expression in renal tissues from type 2 diabetic nephropathy biopsies were increased, supporting the presence of increased cellular senescence in DN [23,24]. A high-glucose environment has been demonstrated to induce the senescence of renal tubular epithelial cells, glomerular mesangial cells, endothelial cells, and podocytes [25,26]. The accumulation of senescent cells and their associated SASP is a major driving factor for chronic kidney disease (CKD) and renal aging [27]. In recent years, novel therapeutic approaches have been developed to prevent senescence-related organ dysfunction by targeting senescent cells [28]. Senolytic agents are reagents that can selectively induce the death of senescent cells. Experimental evidence has shown that such drugs can reverse the senescent phenotype and improve renal function [29,30]. Senescence plays an important role in the field of kidney disease treatment. However, the link between DN and cellular senescence is not yet fully understood. Therefore, elucidating the role of cellular senescence in the pathogenesis and progression of DN is critical.

In this study, we employed bioinformatics methods, such as differential expression analysis, weighted gene co-expression network analysis (WGCNA), machine learning algorithms, and protein–protein interaction (PPI) networks, to process the transcriptomic data of diabetic nephropathy. This approach has enabled the identification of key pathogenic genes associated with cellular senescence. Further analysis explored the relationship between these key genes and immune cell infiltration. Single-cell data were used to determine the cellular localization of these critical genes. Finally, in vitro experiments were performed to validate the accuracy of the identified genes. These novel genes may significantly enhance the diagnosis and treatment of DN and provide potential targets for precise prevention and personalized therapeutic strategies.

## 2. Results

### 2.1. Identification of DEGs

**Figure 1:**
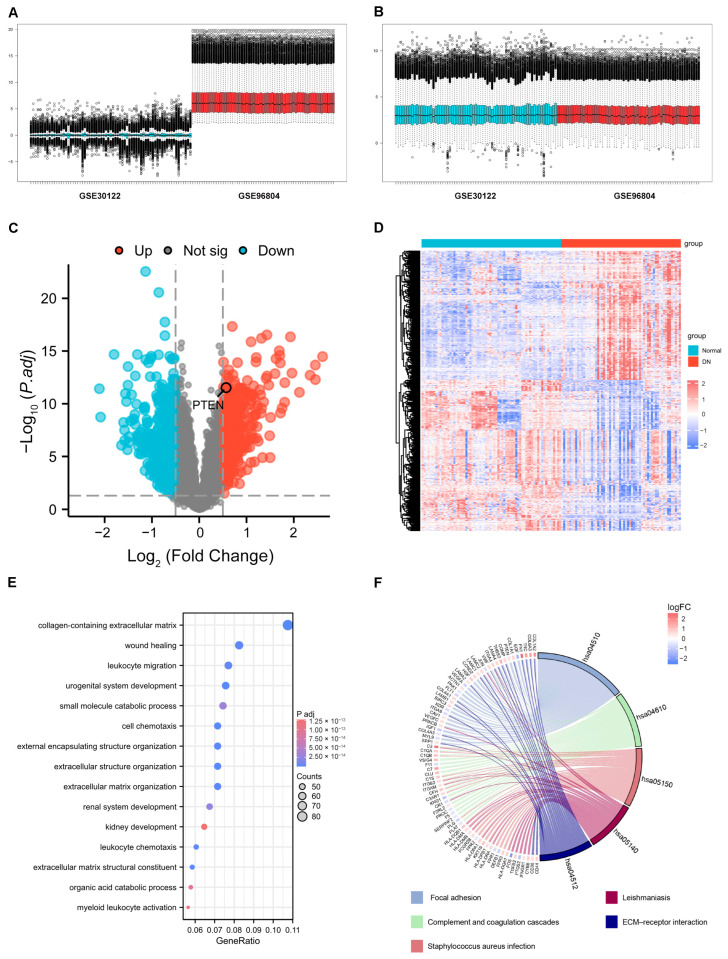
Identification of DEGs in training set (GSE96804 and GSE30122). (**A**) Evaluation of the batch effect before merging training set. The samples of GSE30122 are in blue, and the samples of GSE96804 are in red. (**B**) Evaluation of the batch effect after merging training set. The samples of GSE30122 are in blue, and the samples of GSE96804 are in red. (**C**) Volcano plot of DEGs in training set. Red and blue represent DEGs with significantly higher and lower expression level in DN groups, respectively. (**D**) Heatmap of expression of DEGs. (**E**) Gene ontology enrichment analysis of DEGs. (**F**) KEGG enrichment analysis of DEGs.

GSE96804 and GSE30122 were merged into the training set (Figure 1A). We obtained a dataset of 70 normal and 60 DN samples after removing the batch effect (Figure 1B). After analyzing the training set, we identified 761 DEGs, comprising 351 upregulated and 410 downregulated genes. Figure 1C,D depict the volcano plot and heatmap of DEGs, respectively. We performed GO and KEGG enrichment analyses of DEGs. GO analysis reveals that these genes are associated with the collagen-containing extracellular matrix, wound healing, and leukocyte migration (Figure 1E). KEGG enrichment analysis suggests that these genes are involved in focal adhesion, complement and coagulation cascades, Staphylococcus aureus infection, leishmaniasis, and ECM-receptor interaction (Figure 1F).

### 2.2. The Result of Weighted Gene Co-Expression Network Analysis

**Figure 2:**
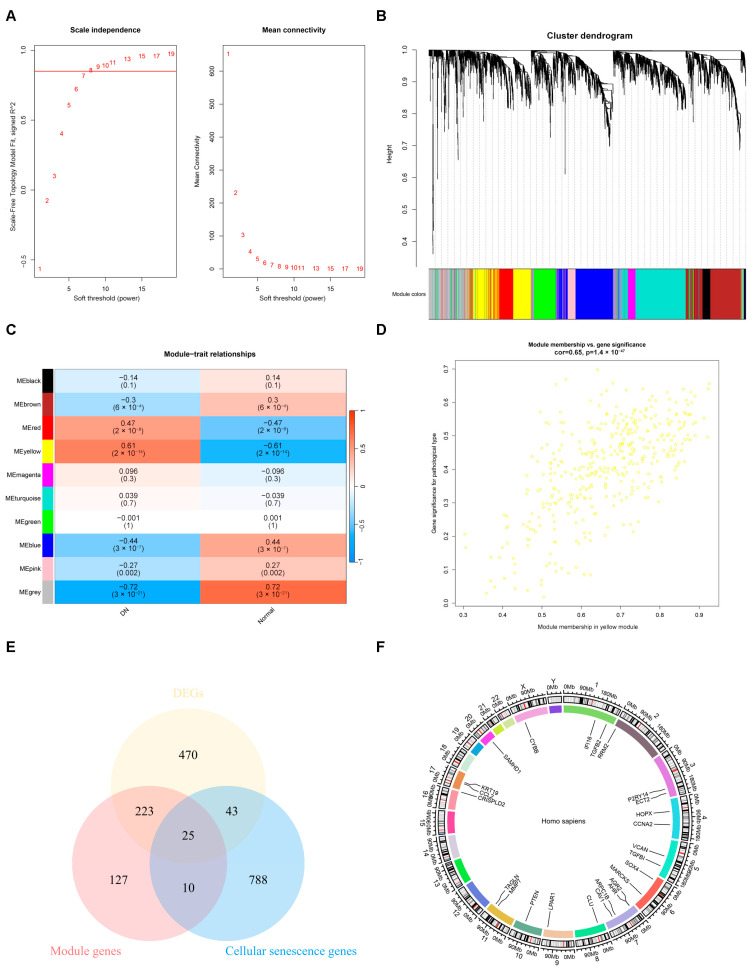
Identification of hub modules using the WGCNA analysis. (**A**) Scale-free R^2^ value is 0.85 (red line). β = 8 is selected as the soft threshold with the combined analysis of scale independence and mean connectivity. (**B**) Gene hierarchy tree-clustering diagram. Different colors represent different module genes. (**C**) Heatmap of correlation between module genes and phenotypes. (**D**) Scatter plot of the yellow module. (**E**) A Venn diagram of the module genes, DEGs, and cellular senescence-related genes. (**F**) Chromosome localization map of intersecting genes.

WGCNA was used to identify the modules most strongly associated with DN. We chose β = 8 (scale-free R^2^ = 0.85), based on the scale independence and mean connectivity (Figure 2A). Ten co-expression gene modules (GCMs) were generated and distinguished using different colors (Figure 2B). The correlation between DN and GCMs was calculated (Figure 2C) using the yellow modules that were selected for subsequent analyses. Figure 2D shows the correlation between gene significance and the clinical traits in the yellow module. Intersection genes (25 genes) of the module genes, DEGs, and cellular senescence-related genes were obtained (Figure 2E). The chromosomal locations of the intersecting genes are visualized and compared in Figure 2F. In addition to *CYBB*, which is located on the X chromosome, the remaining intersecting genes are located on autosomes.

### 2.3. Identification of Potential Subtypes of DN

**Figure 3:**
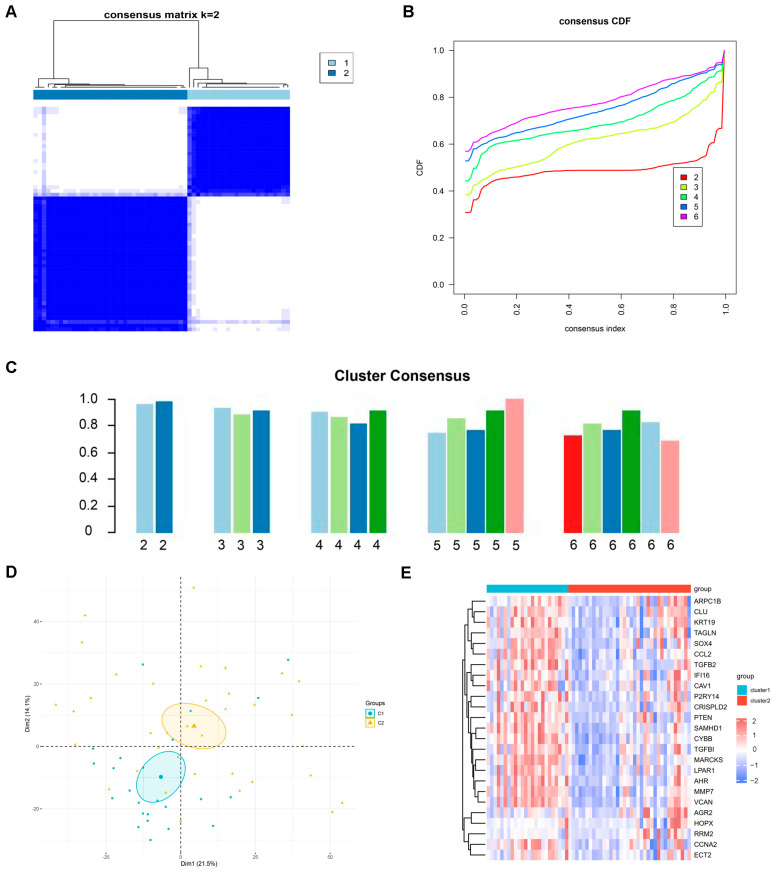
Identification of potential molecular subtypes of DN based on intersecting genes. (**A**) Consensus matrix heatmap when k = 2. (**B**) Representative cumulative distribution function (CDF) curves. (**C**) The score of consensus clustering. (**D**) Principal component analysis plot visualizing the distribution of two clusters. (**E**) Heatmap of intersecting genes in two clusters.

When the k value was set to 2 (k = 2), clustering was the most stable (Figure 3A) and the CDF curve fluctuated at a minimum at k = 2 (Figure 3B). Only when k = 2 was the consistency score for each subtype >0.9 (Figure 3C). Therefore, we divided the 60 DN samples into two clusters, Cluster1 (n = 24) and Cluster2 (n = 36). In addition, PCA revealed significant differences between the two clusters (Figure 3D). The expression of intersecting genes in the two clusters is shown in Figure 3E.

### 2.4. Identification Diagnosis Genes Through Machine Learning and Validation

**Figure 4:**
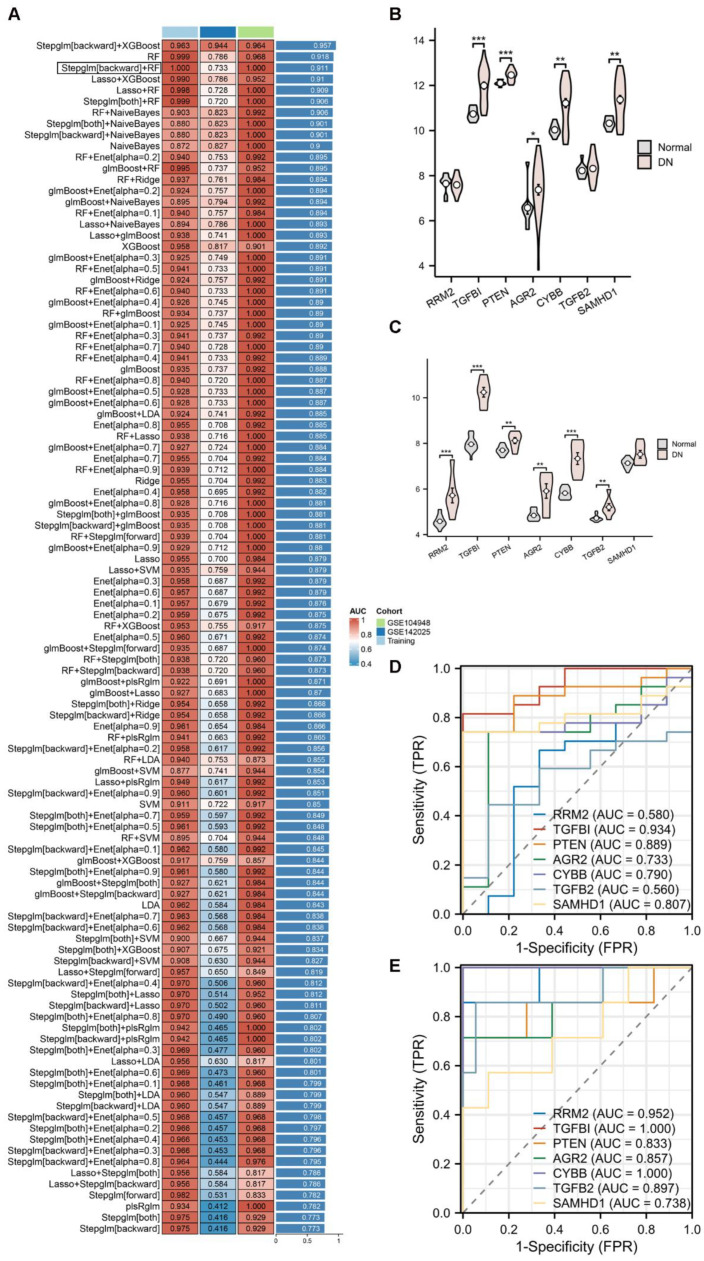
Construction of the diagnostic model. (**A**) Construction of the diagnostic model through a combination of 12 machine learning algorithms. The model was ordered using the average of the AUC of all datasets. The model (“Stepglm[backward]+RF”) in the black box achieved an AUC value of 1 in the training set and was identified as the optimal model, comprising seven genes (*RRM2*, *TGFB1*, *PTEN*, *AGR2*, *CYBB*, *TGFB2*, and *SAMHD1*). (**B**) Expression of diagnostic genes in validation set GSE142025. *, *p <* 0.05, **, *p* < 0.01, ***, *p* < 0.001. (**C**) Expression of diagnostic genes in validation set GSE104948. **, *p* < 0.01, ***, *p* < 0.001. (**D**) ROC curve of diagnostic genes in validation set GSE142025. (**E**) ROC curve of diagnostic genes in validation set GSE104948.

We employed 12 commonly used machine learning algorithms to identify the optimal diagnostic genes, which were applied to both the training and validation sets (GSE104948 and GSE142025) to determine the best model. The “Stepglm[backward]+RF” model achieved an AUC value of 1 in the training set and was identified as the optimal model, comprising seven genes (*RRM2*, *TGFB1*, *PTEN*, *AGR2*, *CYBB*, *TGFB2*, and *SAMHD1*) (Figure 4A). Figure 4B shows the expression of the seven genes in GSE142025, in which the expression levels of *RRM2* and *TGFB2* did not differ between the DN and normal groups. Figure 4C shows the expression of seven genes in GSE104948, with no significant difference in *SAMHD1* expression between the DN and normal groups. Figure 4D,E show the ROC curves of the seven genes in GSE142025 and GSE104948, respectively. Ultimately, four genes were identified as the best diagnostic markers: *TGFB1*, *PTEN*, *AGR2*, and *CYBB*.

### 2.5. PPI Network Construction and Important Module Analysis

**Figure 5:**
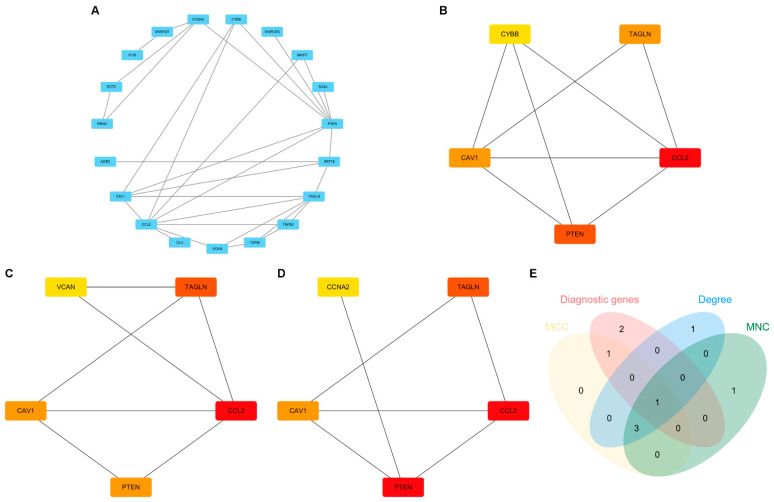
Screening hub genes by PPI network. (**A**) PPI network. (**B**) The top 5 genes based on MCC. (**C**) The top 5 genes based on MNC. (**D**) The top 5 genes based on Degree. (**E**) A Venn diagram of the genes screened from MCC, MNC, Degree, and diagnostic genes.

We constructed a PPI network (Figure 5A) using 25 intersection genes and employing MCC, MNC, and Degree algorithms to identify hub genes (Figure 5B–D). In total, only *PTEN* was designated as a hub gene (Figure 5E).

### 2.6. The Result of Immune Infiltration Analysis

**Figure 6:**
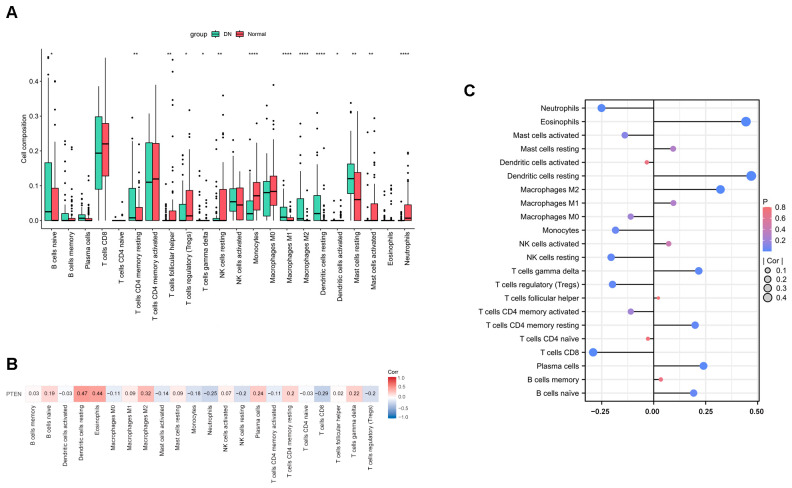
Immune cell infiltration analysis. (**A**) Comparison of infiltrating immune cells in DN and normal samples. *, *p* < 0.05, **, *p* < 0.01, ****, *p* < 0.0001. Student’s *t*-test. (**B**) Correlation analysis of *PTEN* and immune cells. Spearman correlation analysis. (**C**) Visualization of the correlation between *PTEN* and immune cells.

Using the CIBERSORT algorithm, the proportion of ICI in DN and normal tissues was calculated. The box plot showed a significant increase in the infiltration of M1 macrophages (*p* < 0.0001), M2 macrophages (*p* < 0.0001), and resting dendritic cells (*p* < 0.0001), while there was a decrease in the infiltration of monocytes (*p* < 0.0001) and neutrophils (*p* < 0.0001) in DN samples compared to normal tissues (Figure 6A). To determine whether *PTEN* plays an important role in DN pathogenesis by regulating immune cells, the correlation between *PTEN* expression and immune cells was further analyzed (Figure 6B). The results show that *PTEN* is positively correlated with M2 macrophages (r = 0.32, *p* = 0.0002) and resting dendritic cells (r = 0.47, *p* = 1.81 × 10^−8^) but inversely correlated with monocytes (r = −0.18, *p* = 0.036) and neutrophils (r = −0.25, *p* = 0.004) (Figure 6C).

### 2.7. The Study of Disease Gene Expression Levels

**Figure 7:**
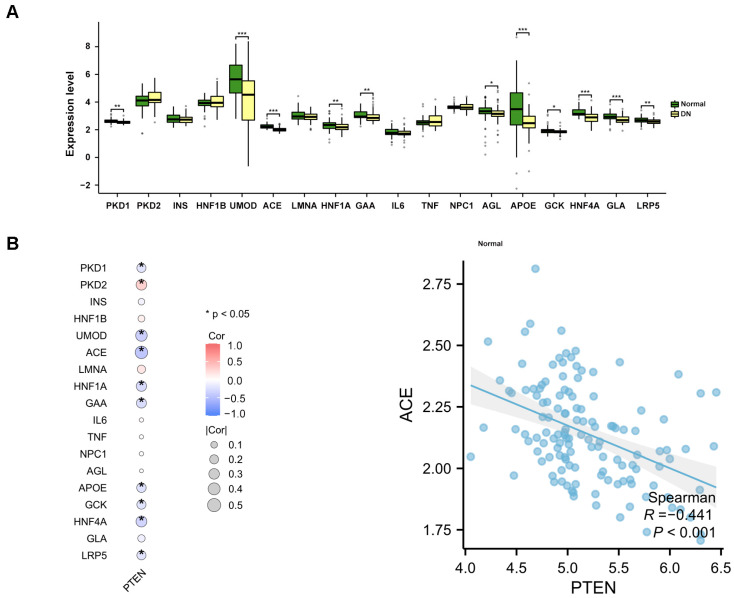
Connection between the level of *PTEN* and several DN-related genes. (**A**) Box plots displaying the expression of the top 20 genes related to DN. *, *p* < 0.05, **, *p* < 0.01, ***, *p* < 0.001. (**B**) Correlation between the level of *PTEN* and the expression of several DN-related genes. *, *p* < 0.05.

DN-related genes were obtained from the GeneCards database (https://www.genecards.org/) on 2 October 2024 and the top 20 genes with the highest relevance scores were selected for analysis. Among them, *PKHD1* and *KCNJ11* were only present in either GSE96804 or GSE30122, and were thus excluded after merging the training sets. Therefore, 18 genes are included in this analysis. The expression levels of the 18 DN-related disease genes in the training set are shown in Figure 7A. *PTEN* expression significantly correlates with the expression levels of several disease-related genes. Notably, compared to the normal group, *ACE* expression was reduced in DN, and *PTEN* expression was negatively correlated with *ACE* expression (r = −0.441, *p* < 0.001) (Figure 7B), indicating that high expression of *PTEN* is positively correlated with the incidence rate of DN. This result is consistent with the findings of previous studies.

### 2.8. Annotation and Analysis of Cluster Subtypes of Single-Cell Data

**Figure 8:**
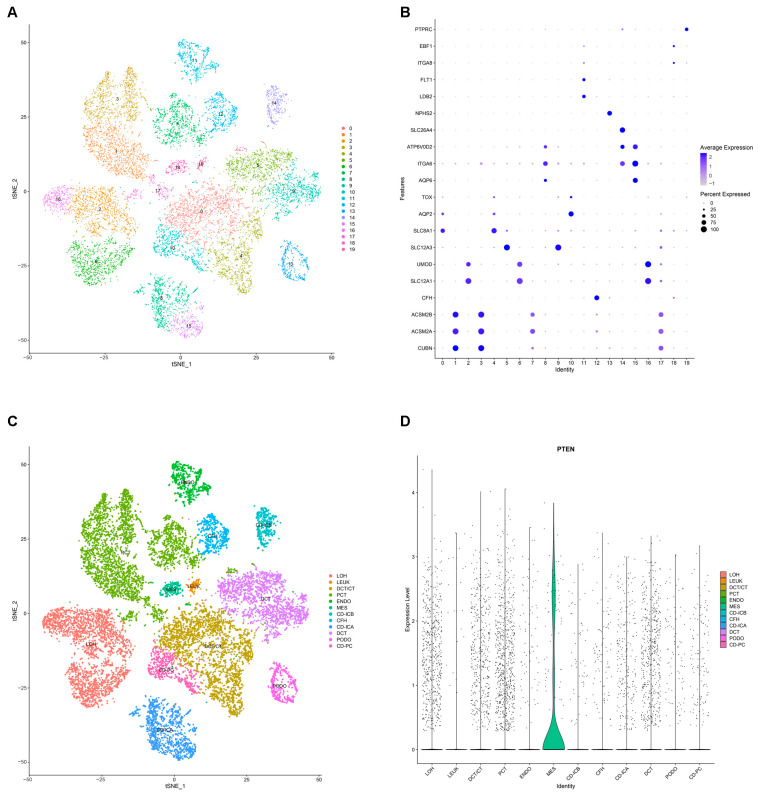
Single Nucleus RNA Sequencing. (**A**) Annotation of cluster subtypes. (**B**) Cell clusters are recognized by renal cell type-specific DEGs. (**C**) tSNE plots of cell clusters. (**D**) The expression distribution of *PTEN*.

Cells were filtered based on RNA counts between 200 and 30,000, RNA features between 200 and 5000, mitochondrial RNA content less than 20%, and hemoglobin RNA content less than 5%, resulting in 21,272 cells and 15,594 genes for further analysis. The tSNE algorithm was used to group cells into 19 distinct clusters (Figure 8A). Figure 8B shows the expression of kidney-specific genes across 19 clusters. Ultimately, 19 clusters were annotated into 12 cell types (Figure 8C): Loop of Henle (LOH), leukocytes (LEUK), distal convoluted tubule/connecting tubule (DCT/CT), proximal convoluted tubule (PCT), endothelium (ENDO), mesangial cells (MES), collecting duct–intercalated cell A (CD-ICA), collecting duct–intercalated cell B (CD-ICB), complement factor H (CFH), distal convoluted tubule (DCT), podocytes (PODO), and collecting duct principal cells (CD-PC). *PTEN* expression is visualized using a violin plot, which shows that *PTEN* is predominantly expressed in the mesangial cells (Figure 8D).

### 2.9. Validation of the Expression of PTEN

**Figure 9:**
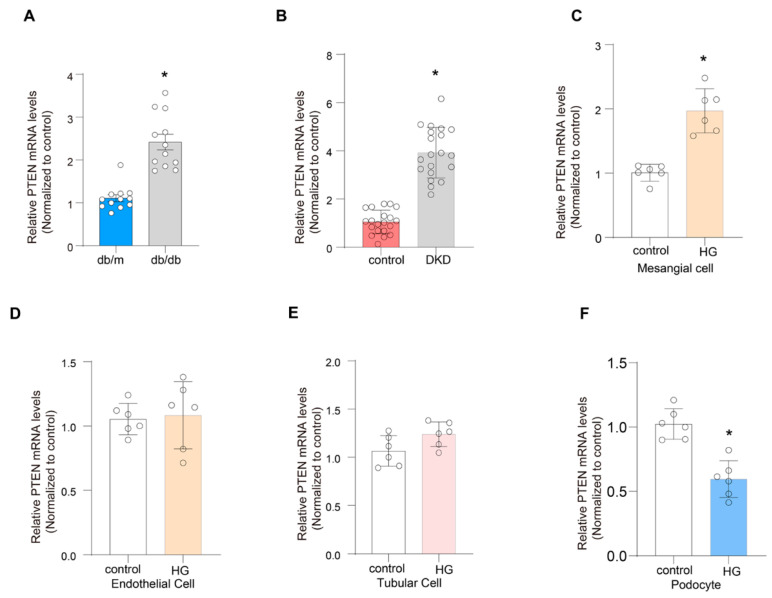
Validation of the expression of *PTEN*. (**A**) The relative expression levels of *PTEN* mRNA in the kidney of different groups of *mice*. Data are expressed as mean ± SEM. * *p* < 0.05 using two-tailed Student’s unpaired *t*-test analysis. (**B**) The relative expression levels of *PTEN* mRNA in the kidney of *human* renal biopsy. Data are expressed as mean ± SEM. * *p* < 0.05 using two-tailed Student’s unpaired *t*-test analysis. (**C**–**F**) *PTEN* mRNA levels in high-glucose-treated cultured mesangial cells, endothelial cells, tubular cells, and podocytes. Data are expressed as mean ± SEM. * *p* < 0.05 using two-tailed Student’s unpaired *t*-test analysis.

The mRNA expression level of *PTEN* was elevated in tissues from db/db *mice* (Figure 9A). The mRNA expression was also upregulated in kidneys from patients with DN (Figure 9B). Additionally, high-glucose treatment significantly increased *PTEN* mRNA levels in cultured mesangial cells, while *PTEN* expression was downregulated in podocytes (Figure 9C–F).

## 3. Discussion

Diabetic nephropathy (DN) is one of the microvascular complications of type II diabetes and a major cause of end-stage renal disease (ESRD) [31,32], imposing a significant burden on healthcare systems and society [2]. The pathogenesis of DN is highly complex, but studies have indicated that cellular senescence plays a role in its onset and progression [19,33]. Cellular senescence in DN involves multiple mechanisms, including telomere shortening, DNA damage, and epigenetic modifications [33]. Telomeres, which are conserved tandem nucleotide repeats, play a critical role in maintaining genomic integrity [34]. Loss of telomeres has been associated with renal cell senescence and the progression of DN. In a hyperglycemic environment, oxidative stress and the accumulation of advanced glycation end-products (AGEs) lead to DNA damage, promoting the premature senescence of renal cells [35]. Hyperglycemia also induces macrophages to secrete senescence-associated secretory phenotypes (SASPs), triggering chronic inflammation and directly promoting the senescence of mesangial cells and tubular epithelial cells [26,36]. Epigenetic modifications, including cytosine DNA methylation, post-translational modifications (PTMs) of histones, and non-coding RNAs, regulate the interaction between genes and the intracellular environment. These modifications influence gene expression and function without altering the DNA sequence itself [37]. Experimental evidence supports a relationship between DNA methylation and the normal function of glomerular and proximal tubular epithelial cells [38]. A study has also shown reduced DNA methylation of *MIOX* in the kidneys of DN *mice* and in tubular cells cultured under high-glucose conditions [39]. Owing to the fact that cell senescence plays a crucial pathogenic role in DN, the development of novel senescence-related markers for DN diagnosis and treatment is significant. In this study, we downloaded DN-related datasets from the GEO database and identified key senescence-related genes involved in DN through differential expression analysis and WGCNA. To identify the most diagnostically significant genes, we combined 12 machine learning algorithms to construct diagnostic models for the first time. After validation, *TGFB1*, *CYBB*, *PTEN*, and *AGR2* were identified as the diagnostic genes. Subsequently, we constructed a PPI network and, using MCC, MNC, and Degree algorithms, *PTEN* was identified as the most critical senescence-related gene involved in DN. Compared to normal tissues, *PTEN* was upregulated in DN, which was further validated by the experimental results.

*PTEN* (phosphatase and tensin homolog) is a dual-function lipid and protein phosphatase that regulates various cellular processes, including cell growth, migration, and metabolism, through the PI3K/Akt signaling pathway [40,41,42]. As a well-established tumor suppressor gene, it has been extensively studied and confirmed to play a role in multiple pathological processes [43]. Loss of *PTEN* promotes podocyte cytoskeleton rearrangement and exacerbates DN [42]. miRNAs, such as miR-1297 [44] and miR-21 [45], regulate DN progression by targeting *PTEN*. *PTEN*-induced serine/threonine kinase 1 (PINK1) plays a protective role against DN by alleviating mitochondrial dysfunction and necroptosis [46]. These findings suggest that *PTEN* plays a role in DN development. In this study, we found that the expression of *PTEN* in mesangial cells cultured under high-glucose conditions was higher than that in cells cultured under low-glucose conditions, consistent with the results of Zhang et al. [47]. However, other studies have shown that *PTEN* expression in mesangial cells decreases significantly after 48 h of high-glucose stimulation compared with that in the normal control group [48]. Activation of the PI3K/Akt signaling pathway can induce endoplasmic reticulum stress and activate the NF-κB signaling pathway, ultimately leading to apoptosis under high-glucose conditions [49]. Therefore, we hypothesized that the protective effect of *PTEN* could be achieved through negative regulation of the PI3K/Akt pathway. During the progression of DN, the PI3K/Akt signaling pathway is excessively activated [50]. As a lipid phosphatase, *PTEN* can negatively regulate this pathway through its high expression, thereby improving podocyte phenotype and alleviating podocyte injury [51]. However, owing to continuous stimuli, such as oxidative stress, inflammatory responses, and mitochondrial dysfunction, the protective effect of *PTEN* weakens over time, and its expression ultimately decreases as kidney damage worsens. Compounds such as non-toxic AS101 have been shown to slow DN progression and are associated with decreased p-AKT and increased *PTEN* levels [52]. miR-214 may represent a novel therapeutic target for DN. The level of miR-214 in the renal cortex of diabetic *mice* was significantly elevated. It was found, through in vivo and in vitro experiments, that the inhibition of miR-214 could restore the level of *PTEN* in *human* mesangial cells stimulated by high levels of glucose, and the cross talk between miR-214 and *PTEN* alleviated glomerular hypertrophy [53]. Chen’s research revealed that oleanolic acid upregulated the expression of *PTEN* by targeting miR-142 and the PI3K/Akt/mTOR pathway in NRK-52E cells, thereby alleviating mesangial cell injury [54]. It has been found that the overexpression of *PTEN* could antagonize the autophagy inhibition and the expression of Collagen IV induced by high levels of glucose and miR-22, leading to a reduction in fibrosis [55]. Wang et al. discovered that the overexpression of *PTEN* in podocytes could protect the kidneys from the impact of hyperglycemia in vivo, suggesting that targeting *PTEN* may be a novel and promising therapeutic strategy for the treatment of DN [56]. Genetic intervention targeting *PTEN* has emerged as a promising approach for the treatment of acute kidney injury and chronic kidney disease. Administration of 3-Deazaneplanocin A (DZNep) has been shown to effectively inhibit the specific binding of *EZH2* to the proximal promoter of *PTEN*, thereby reducing *PTEN* mRNA transcription levels and ameliorating CKD-associated pathological damage [57]. Additionally, reversing *PTEN* transcriptional repression in highly invasive tumors such as melanoma using the CRISPR system has been demonstrated to effectively suppress cancer cell proliferation and migration [58]. Modulating *PTEN* expression in renal tissues holds potential as a therapeutic strategy for DN. Taken together, we propose that *PTEN* plays a protective role in diabetic nephropathy.

Our study demonstrated a significant increase in both M1 and M2 macrophages in DN, consistent with previously published findings [59]. Renal biopsies from patients with DN confirmed the presence of macrophages in both the glomerular and interstitial regions at all stages of DN [60,61]. TNF-α plays a pivotal role in the progression of DN, with markedly elevated expression levels observed in DN animal models [62,63]. Pharmacological inhibition of TNF-α leads to reduced kidney injury in DN patients [64]. Macrophages serve as a major source of TNF-α [65], and TNF-α expression in DN animal models decreases upon TNF-α knockout in macrophages [66]. Cellular senescence is characterized by the production of senescence-associated secretory phenotype (SASP) components. Macrophages are key SASP-mediating cells that potentially contribute to the maintenance of the SASP response within the renal microenvironment, thereby promoting chronic inflammation [67,68,69]. M1 macrophages are characterized by the production of proinflammatory cytokines, and the subsequent inflammatory response may lead to organ dysfunction [70]. In contrast, M2 macrophages are associated with the production and secretion of anti-inflammatory cytokines, which alleviate inflammation [71]. Akt is a key protein that promotes M1 polarization, and its phosphorylation enhances M1 macrophage polarization while inhibiting M2 polarization, thereby promoting inflammation [72]. *PTEN* converts PI (3,4,5) P3, which plays a central role in Akt phosphorylation, into PI (4,5) P2, thereby negatively regulating the PI3K/Akt signaling pathway and inhibiting Akt phosphorylation, leading to increased M2 polarization [73]. This is consistent with our findings, that show a positive correlation between *PTEN* and M2 macrophages. Inhibition of M1 macrophage activation and the promotion of M2 macrophage transformation can prevent podocyte injury [74], and the anti-inflammatory effects of M2 macrophages play a role in resisting cellular senescence. In summary, these findings further support the protective role of *PTEN* in early diabetic nephropathy.

## 4. Materials and Methods

### 4.1. Human Renal Biopsy Samples

Renal biopsies were performed as part of the routine clinical diagnostic investigation and were obtained from the Department of Pathology, Qilu Hospital, Shandong University. Normal control samples were obtained from the healthy kidney poles of individuals without kidney disease who underwent tumor nephrectomy. Relevant clinical information is included in Supplementary Table S1. The investigations were conducted in accordance with the principles of the Declaration of Helsinki and were approved by the Research Ethics Committee of Shandong University after informed consent had been obtained from all patients.

### 4.2. Animal Studies

All experimental protocols for animal studies were approved by the Institutional Animal Care and Use Committee of the School of Basic Medical Sciences, Shandong University, and were conducted in accordance with the National Institutes of Health Guide for the Care and Use of Laboratory Animals. db/db and db/m *mice* were purchased from the Beijing Vital River Laboratory Animal Technology Co., Ltd. (Beijing, China). All *mice* (3-5 *mice* per cage) were housed under standard laboratory SPF conditions with ad libitum access to water and standard laboratory chow diet (Beijing KEAOXIELI Feed Company, Beijing, China). Water and cages were autoclaved. Cages with standard corncob bedding were changed three times per week. Littermate control *mice* were used for all in vivo experiments. All experimental animals were kept under barrier conditions with constant veterinary supervision, and did not display signs of distress or pathological changes that warranted veterinary intervention.

### 4.3. Cell Culture and Treatment

*Human* glomerular mesangial cells were obtained from the American Type Culture Collection (Cell Systems, Kirkland, WA, USA) and cultured in RPMI 1640 medium with 10% FBS. *Human* glomerular endothelial cells were obtained from Sciencell Research Laboratories (Carlsbad, CA, USA) and cultured in RPMI 1640 medium with 10% FBS. *Human* podocytes and tubular epithelial cells were kindly gifted by Professor Chun Zhang, Tongji Medical College, Huazhong University of Science and Technology. The use of the gifted cell lines was approved by the Research Ethics Committee of Shandong University. The high-glucose (Sigma-Aldrich, Beijing, China) concentration was 35 mmol/L.

### 4.4. Data Sources

The search formula “(“diabetic nephropathies”[MeSH Terms] OR Diabetic Nephropathy[All Fields]) AND “*Homo sapiens*”[porgn] AND ((“Expression profiling by high throughput sequencing”[Filter] OR “Expression profiling by array”[Filter]) AND (“2014/01/01”[PDAT]: “2023/12/31”[PDAT]))” was used to identify appropriate gene expression datasets in the GEO database (http://www.ncbi.nlm.nih.gov/geo) on 10 August 2024. Finally, the GSE96804, GSE142025, GSE104948, and GSE131882 datasets were included in this study. In addition, GSE30122 was also included in this study due to the large sample size (69 samples). GSE96804 and GSE30122 were designated as the training sets, whereas GSE142025 and GSE104948 served as the validation sets. GSE96804 was downloaded from the GPL17586 platform (HTA-2_0 Affymetrix *Human* Transcriptome Array 2.0 [transcript (gene) version]) and included 20 normal and 41 DN samples. GSE30122 was sourced from the GPL571 platform ([HG-U133A_2] Affymetrix *Human* Genome U133A 2.0 Array) and contained 50 normal and 19 DN samples. GSE142025 was obtained from the GPL20301 platform (Illumina HiSeq 4000 (*Homo sapiens*)) and comprised 9 normal samples and 27 DN samples. GSE104948 was downloaded from the GPL22945 platform ([HG-U133_Plus_2] Affymetrix *Human* Genome U133 Plus 2.0 Array [CDF: Brainarray HGU133Plus2_Hs_ENTREZG_v19]), with 18 normal samples and 7 DN samples. The GSE131882 dataset contained snRNA data from three normal samples and three DN samples downloaded from GPL24676 (Illumina NovaSeq 6000 (*Homo sapiens*)). Genes related to cellular senescence were sourced from the GenAge database (https://genomics.senescence.info/genes/index.html) on 10 August 2024. The clinical information for the training set can be found in the study by Shi [75] and Woroniecka [76].

### 4.5. Identification of Differentially Expressed Genes and Functional Enrichment Analysis

We used the sva package (version 3.50.0) in the R software (version 4.3.2) to remove batch effects from the training sets (GSE96804 and GSE30122). The limma package (version 3.58.1) was used for differential analysis, defining genes with adjusted *p* value < 0.05 and |logFC| ≥ 0.5 as differentially expressed genes (DEGs), while the ggplot2 (version 3.5.1) and pheatmap packages (version 1.0.12) were used to generate volcano plots and heatmaps of DEGs, respectively.

GO analysis helps to better understand the functional annotation of gene products [77], while KEGG pathways analysis not only facilitates the annotation of the function of the genes themselves, but also that of genes involved in various signaling pathways [78]. DEGs were subjected to GO and KEGG enrichment analyses using the clusterProfiler package [79] (version 4.10.1) in the R software (version 4.3.2), and visualization was performed using the ggplot2 package (version 3.5.1). *FDR* < 0.05 and a gene count ≥ 5 was considered statistically significant.

### 4.6. Weighted Gene Co-Expression Network Analysis

WGCNA is a method that studies the associations between genes and phenotypes by constructing gene co-expression networks [80]. A weighted gene co-expression network was constructed using the WGCNA package [80] (version 1.73) in the R software (version 4.3.2) to explore the relationship between genes and phenotypes. Initially, the top 25% of genes with the highest variance was selected for further analysis. The soft-thresholding power was set to 8, achieving a scale-free R^2^ value of 0.85. The weighted adjacency matrix was then transformed into a topological overlap matrix (TOM), with “TOMType” set to “unsigned”. To identify modules, “minModuleSize” of 50 was applied, and modules were detected based on the TOM-derived dissimilarity metric (1-TOM) using the hierarchical clustering tree algorithm. Each module was assigned a unique color. Among the 10 gene modules identified, the yellow module was selected for further analysis. A Venn diagram was used to identify intersecting genes among the DEGs, yellow module genes, and cellular senescence-related genes. Finally, the circlize package (version 0.4.16) was used to generate a chromosomal location map of intersecting genes.

### 4.7. Clustering Analysis to Identify the Potential Subtypes of DN

Consensus clustering, which serves as a means to identify molecular subtypes predicated on an approximate quantity of clusters, was employed. Through the application of the k-means method, it enabled the discovery of DN subgroups [81]. Based on the expression profiles of intersecting genes strongly associated with DN, the ConsensusClusterPlus package [82] (version 1.66.0) was used to perform consensus clustering, revealing distinct DN subtypes. The maximum number of clusters (maxK) was set to 6, allowing for exploration of clustering solutions ranging from 2 to 6 clusters, to comprehensively capture the data structure. The Pearson correlation was used as the distance metric, considering the numerical nature of the data and the potential linear relationships among variables, thereby providing a robust measure of sample similarity. To ensure the stability and reproducibility of the results, the number of repetitions (reps) was set to 50, minimizing the impact of randomness. Principal component analysis (PCA) was conducted using the scatterplot3d (version 0.3.44) and factoextra packages (version 1.0.7) to visualize the clustering results.

### 4.8. The Construction of a PPI Network

To explore protein interactions, pathways, and co-expression relationships, we used the STRING database (https://cn.string-db.org/) on 10 August 2024 to construct a key protein–protein interaction network for cellular senescence-related genes identified via differential analysis and WGCNA. The minimum required interaction score was set at 0.400. The network was refined using the Cytoscape software (www.cytoscape.org/) (version 3.10.0) on 10 August 2024, and the cytoHubba plugin was used to identify significantly interacting genes. Hub genes were defined as the intersection of the top 10 genes identified by the Degree, maximal clique centrality (MCC), and maximum neighborhood component (MNC) algorithms.

### 4.9. Diagnostic Genes Identified via Machine Learning

We integrated 12 commonly used machine learning algorithms and evaluated 107 combinations of these. These algorithms include least absolute shrinkage and selection operator (Lasso), stepwise generalized linear model (Stepglm), support vector machine (SVM), generalized linear model by likelihood-based boosting (glmBoost), Ridge, Elastic Net (Enet), partial least squares regression for generalized linear models (plsRglm), random forest (RF), linear discriminant analysis (LDA), extreme gradient boosting (XGBoost), generalized boosted regression modelling (GBM), and Naive Bayes. Descriptions of these 12 machine learning algorithms are summarized in Supplementary File S1 [83,84]. Training was performed using the training set, whereas validation was conducted using the GSE104948 and GSE142025 datasets. The area under the curve (AUC) for each cohort, as well as the average AUC, was calculated.

### 4.10. Immune Infiltration Analysis

CIBERSORT is a deconvolution algorithm designed to measure immune cell infiltration (22 different cell types) within the gene expression profile of a training set. The immune cell infiltration outcomes in the samples were visually presented using the ggplot2 package (version 3.5.1). Spearman correlation analysis was performed to ascertain and visualize the correlation between the core gene and infiltrating immune cells using ggcorrplot (version 0.1.4.1) and ggplot2 packages (version 3.5.1).

### 4.11. Single Nucleus RNA Sequencing

The Seurat package [85] (version 5.1.0) in R software (version 4.3.2) was used for GSE131882 data analysis. Cells with mitochondrial RNA content less than 20%, hemoglobin RNA content less than 5%, RNA counts between 200 and 30,000, and RNA features between 200 and 5000 were selected for analysis. The NormalizeData function was used to normalize the data, and the FindVariableFeatures function was used to identify the top 2000 highly variable genes. Dimensionality reduction and clustering were performed using t-distributed Stochastic Neighbor Embedding (tSNE) implemented in the Seurat package (version 5.1.0). The analysis was based on the first 19 principal components (dims = 1:19), which were selected after evaluating the elbow plot to capture the majority of variance in the dataset. Clustering was performed using the FindNeighbors and FindClusters functions in Seurat, with a resolution parameter set to 1. This resolution provided an optimal balance between identifying distinct clusters and preserving biological interpretability. Cell annotation was performed using kidney-specific differentially expressed genes [86]. Finally, gene expression was visualized using violin plots.

### 4.12. Real-Time RT-PCR

Real-time quantitative RT-PCR was performed using the Ultra SYBR Mixture (Nuoyang Biotechnology, Hangzhou, China). The mRNA levels for target genes were analyzed using a Bio-Rad iCycler system. Levels of the housekeeping gene β-actin were used as an internal control for the normalization of RNA quantity and quality differences among the samples. We calculated fold changes in gene expression normalized to β-actin with the ΔΔCT method using the equation 2-ΔΔCT. *PTEN* primer sequence for *mouse*: Forward TGAGTTCCCTCAGCCATTGCCT; Reverse GAGGTTTCCTCTGGTCCTGGTA. *PTEN* primer sequence for *human*: Forward TGAGTTCCCTCAGCCGTTACCT; Reverse GAGGTTTCCTCTGGTCCTGGTA.

### 4.13. Quantification and Statistical Analysis

Data are expressed as mean ± SEM. Statistical analyses were performed with GraphPad Prism (version 8.0, GraphPad Software, San Diego, CA, USA). Bioinformatics analyses were performed with R software (version 4.3.2). Comparisons between two groups were performed using the two-tailed Student’s *t*-test for normally distributed data and the Mann–Whitney rank sum test for non-normally distributed data. Differences between multiple groups with one variable were determined using one-way ANOVA followed by a post hoc Tukey’s test. Different groups of *mice* were allocated in a randomized manner, and investigators were blinded to the allocation of different groups when carrying out surgeries and outcome evaluations. Exclusion criteria were based on animal well-being at the beginning of the study. No power analysis was performed to determine the sample size. The sample size in each study was based on experience with previous studies in our lab.

## 5. Conclusions

This study identified *PTEN* as a potential diagnostic biomarker for DN, offering promise for early detection and personalized therapeutic strategies. These findings provide valuable insights into the role of cellular senescence in DN pathogenesis.

## Data Availability

All data generated or analyzed in this study are included in the main text and the Supplementary Materials for this article. Other source data that support the findings of this study are available from the corresponding author upon reasonable request.

## Supplementary Materials

The following supporting information can be downloaded at: https://www.mdpi.com/article/10.3390/ijms26073088/s1.
