# Supplementary material for: PTEN: A Novel Diabetes Nephropathy Protective Gene Related to Cellular Senescence
Source: Int J Mol Sci. 2025 Mar 27;26(7):3088. doi: 10.3390/ijms26073088 (PMC11988946; doi:10.3390/ijms26073088)
Supplement: Supplementary file 1 [file ijms-26-03088-s001.zip › Supplementary File.pdf]

Description of Machine Learning Algorithms Used

To identify diagnostic genes, we employed 12 machine learning algorithms, as detailed below:

1.Lasso, Ridge, and Enet

Lasso applies L1 regularization to linear regression models, effectively performing feature selection by penalizing the absolute size of regression coefficients. It is particularly useful for addressing multicollinearity and reducing overfitting. Ridge employs L2 regularization, penalizing the square of coefficients to address multicollinearity. Unlike Lasso, it does not perform feature selection. Elastic Net combines L1 and L2 penalties, balancing feature selection (L1) and multicollinearity handling (L2). The Lasso, Ridge, and Enet were implemented via the glmnet package, with the optimal regularization parameter (lambda) and the L1-L2 parameter (α, ranging from 0 to 1 in increments of 0.1) tuned using 10-fold cross-validation.

2.Stepglm

Stepglm utilizes stepwise selection (based on AIC/BIC) to identify optimal features for generalized linear models. The glm package in R was used to construct Stepglm models, with stepwise selection directions set to forward, backward, and both.

3.SVM

SVM is a supervised learning model that finds a hyperplane to classify data points while maximizing the margin between classes. It supports nonlinear data using kernel functions. SVM was implemented using the e1071 package in R, with a radial kernel and a cost parameter set to 1.

4.glmBoost

This model integrates gradient boosting with generalized linear models to sequentially minimize residual errors and enhance prediction accuracy. The mboost package in R was used for glmBoost. Hyperparameters were tuned using the cvrisk function with 10-fold cross-validation.

5.LDA

LDA is a linear classification technique that maximizes between-class variance while

minimizing within-class variance. LDA was implemented using the MASS package in R, with cross-validation enabled to compute class probabilities and classifications.

6.plsRglm

This approach combines partial least squares regression with generalized linear models, making it ideal for datasets with high dimensionality and multicollinearity. The plsRglm package in R was used. The cv.plsRglm function with 10-fold cross-validation optimized model performance, with parameters set to pls-glm-logistic for classification tasks.

7.RF

RF constructs multiple decision trees and combines their predictions for classification or regression, improving generalization through feature and sample randomness. The randomForestSRC package in R was used, tuning parameters (mtry and ntree) via grid search with 10-fold cross-validation.

8.GBM

GBM creates a strong learner by sequentially adding weak learners (e.g., decision trees) and correcting previous residuals at each step. GBM was implemented using the gbm package in R. The cv.gbm function optimized the number of trees via 10-fold cross-validation.

9.XGBoost

XGBoost is an enhanced GBM variant, incorporating regularization and tree optimization for improved accuracy and efficiency. The xgboost package in R was used, with optimal parameters determined using 10-fold cross-validation.

10. Naive Bayes

Naive Bayes is a probabilistic model assuming feature independence, used for categorical variable classification based on Bayes' rule. Naive Bayes was implemented using the e1071 package in R, with default parameters for simplicity.
